# Impact of an Oral Nutrition Supplement on the Nutritional Status of Stunted and At-Risk of Stunting Children: A Community-Based Intervention Trial

**DOI:** 10.3390/nu18050754

**Published:** 2026-02-26

**Authors:** Sidra A. Al-Talib, Hamid Jan Jan Mohamed, Amal K. Mitra, Hans Van Rostenberghe, Siti Nur Haidar Hazlan, Ilse Khouw

**Affiliations:** 1 Nutrition Programme, School of Health Sciences, Universiti Sains Malaysia, Kubang Kerian 16150, Kelantan, Malaysia; altalib.sidra21@gmail.com; 2 Department of Public Health, Julia Jones Matthews School of Population and Public Health, Texas Tech University Health Sciences Center, Abilene, TX 79601, USA; amal.mitra@ttuhsc.edu; 3 Paediatrics Department, School of Medical Sciences, Universiti Sains Malaysia, Kubang Kerian 16150, Kelantan, Malaysia; hansvro@usm.my (H.V.R.); cthaidar@usm.my (S.N.H.H.); 4 International Medical School, Management and Science University, Persiaran Olahraga, Section 13, Shah Alam 40000, Selangor, Malaysia; 5 FrieslandCampina, Stationsplein 4, 3818 LE Amersfoort, The Netherlands; ilse.tan-khouw@frieslandcampina.com

**Keywords:** children, growth, milk supplementation, community-based intervention, stunting, Malaysia

## Abstract

**Introduction**: Stunting is associated with poor nutritional intake during early childhood. This study evaluated the effect of a daily intake of 510 mL of an oral nutritional supplement for 180 days on linear growth among children with stunting and at-risk of stunting aged 12–36 months. **Methods**: A community-based, single-arm intervention was conducted among 91 children in Kelantan, Malaysia. The children at enrolment had height-for-age Z-scores (HAZs) between <−1.0 SD and >−3 SD based on WHO Growth Standards. Anthropometric measurements were collected at baseline (T0), 90 days (T90; mid-intervention), and 180 days (T180; post-intervention). Nutrient intake was assessed using 24 h dietary recalls, and compliance was monitored via returned empty sachets. **Results**: The mean age of the children at baseline was 26.7 ± 6.6 months (range, 12.9–36.0 months), with 37 (41%) being stunted and 54 (59%) at risk of stunting. After intervention, the linear growth (height-for-age Z-score) was significantly improved over time (*p* < 0.001) in both stunted and at-risk children. A significant time-by-group interaction (*p* = 0.014) indicated differential effects between the stunted and at-risk groups. Post hoc analysis showed HAZ improvements from baseline (T_0_) to 180 days in stunted and at-risk groups (*p* < 0.001), with the stunted group demonstrating a greater mean change in HAZ compared with the at-risk group. The number of stunted children declined by 37.8% (*p* = 0.003). Nutrient intakes of protein, vitamin D, vitamin C, vitamin B-complex, calcium, phosphorus, magnesium, and iron improved significantly. **Conclusions**: A daily intake of 510 mL of oral nutrition supplement improved linear growth and nutrient intake. These findings support the potential of targeted supplementation in addressing child growth faltering and micronutrient inadequacies.

## 1. Introduction

Despite sustained global efforts, stunting remains a critical public health challenge, with numerous countries including Malaysia falling short of achieving the UN Sustainable Development Goal of reducing stunting among children by 2030 [1]. Stunting, defined as a height-for-age z-score (HAZ) less than −2 standard deviations of the World Health Organization (WHO) Child Growth Standards [2], represents chronic undernutrition during the critical early years of life. Multiple interconnected factors contribute to this condition, including poor maternal nutrition, low birth weight, failure of exclusive breastfeeding, inadequate dietary intake, recurrent infections, and poor environmental sanitation, which compound to create long-term nutritional deficiency in children [3].

Globally, stunting affected 150.2 million children under five years of age in 2024, representing 23.2% of this age group [4]. While the global prevalence has declined from 26.4% in 2012, progress remains insufficient to meet the 2030 target of a 40% reduction [5]. Asia continues to bear the highest burden, with 53% of stunted children globally residing in this region, and South Asia alone accounting for 56 million stunted children [4]. The highest stunting prevalence is observed in South-central Asia (36%) and sub-Saharan Africa regions (East Africa 42%; West Africa 36%), where the number of stunted children continues to increase despite global progress [4].

### 1.1. The Malaysian Context

Malaysia presents a particularly concerning profile among middle-income countries in Southeast Asia. According to the National Health and Morbidity Survey (NHMS), the stunting rate in Malaysia increased alarmingly from 17.7% in 2015 to 21.2% in 2022—a trend that contradicts the declining patterns observed in neighboring Southeast Asian nations [6,7]. This 1.2-fold increase in stunting rate in seven years is quite alarming. It positions Malaysia’s stunting prevalence (21.8%) higher than countries such as the West Bank and Gaza (7.4%) and comparable to Iraq (22.6%), despite Malaysia’s significantly higher economic development [8,9].

Geographic disparities within Malaysia are stark. Kelantan, one of the northeastern states, recorded the highest stunting rate at 28.8%, followed by Pahang (26.2%) and Terengganu (23.4%) [6]. These rates substantially exceed Malaysia’s 2030 target of reducing stunting to 14.2%, and the National Plan of Action for Nutrition 2016–2025 target of 11.0% by 2025, indicating a persistent and worsening nutritional crisis that necessitates immediate intervention [9,10]. Importantly, stunting in Malaysia cuts across all socioeconomic strata: even among households with monthly income exceeding RM5000, the prevalence remains 17.4%, and among children of mothers with tertiary education, the rate is 18.6% [11]. This unusual pattern suggests that factors beyond household income and parental education contribute significantly to stunting in the Malaysian context.

### 1.2. Consequences of Stunting

Stunting represents far more than hindered physical growth; it is a comprehensive indicator of persistent undernutrition with profound and enduring effects across the life course. Evidence consistently demonstrates that early-life stunting among children has major effects on future cognitive function, educational attainment, and health outcomes [12]. Stunted children exhibit increased susceptibility to infections, higher functional impairments, elevated mortality risks, and greater vulnerability to chronic diseases during adulthood [12]. The cognitive and educational impacts are particularly concerning: children who recover to a normal height status (HAZ ≥ −1) by age 5 demonstrate cognitive function levels similar to children who were never stunted, emphasizing the critical importance of early intervention [13]. Children who remain stunted, however, face substantially reduced academic performance, shorter adult height, and diminished economic productivity, perpetuating intergenerational cycles of poverty and undernutrition [14].

### 1.3. Dietary Patterns and Nutritional Challenges in Kelantan, Malaysia

The high prevalence of stunting in Kelantan reflects a complex interaction of socioeconomic, dietary, and cultural factors embedded within the state’s community and living conditions. Understanding these determinants is essential for informing targeted and context-specific interventions to address this persistent public health challenge.

Studies examining childhood malnutrition in neighboring states and similar settings in Malaysia demonstrate that stunting is significantly associated with poverty, low household income, and limited access to food security [15].

In Kelantan, where many households depend on agriculture and fishing, economic constraints and seasonal income instability can limit access to diverse, nutrient-dense foods, increasing children’s vulnerability to undernutrition [10]. Dietary patterns dominated by affordable, carbohydrate-based traditional foods may further contribute to inadequate protein and micronutrient intake during critical growth periods [16,17].

Maternal and household factors, including maternal education, household sanitation, and infant feeding practices, have also been identified as important determinants of child growth outcomes in Malaysian populations [17]. These interrelated factors underscore the need for culturally appropriate nutrition interventions that address both dietary quality and underlying socioeconomic constraints in Kelantan.

### 1.4. Nutritional Determinants and Intervention Approaches

Micronutrient deficiencies play a pivotal role in growth faltering. Deficiencies in vitamin A, iron, zinc, and iodine contribute to 35% of deaths in children under five years of age [18]. Inadequate intake of calcium and vitamin D further increases the likelihood of stunting [19]. Recent evidence from systematic reviews demonstrates that prenatal multi-micronutrient supplementation (MMS) produces superior effects compared to single-nutrient approaches in promoting optimal growth in children [20,21]. Multiple micronutrient interventions have shown efficacy in improving not only anthropometric outcomes but also biochemical markers of nutritional status across diverse populations [22].

Evidence from intervention studies conducted in Asian and African settings demonstrates that nutrient-dense oral nutrition supplementation (ONS) can significantly improve height, weight, and growth z-scores in undernourished children [13,23]. Recent randomized controlled trials in Vietnam showed that ONS combined with dietary counseling resulted in significant improvements in height-for-age percentiles, with effects becoming evident after 24 weeks of supplementation, and approximately 40% of stunted children recovering to a normal height status after 6 months [13,24]. Similarly, studies in India demonstrated that ONS with dietary counseling was more effective than counseling alone in promoting catch-up growth, with significant improvements in weight-for-age and height-for-age z-scores [23,25].

Linear growth has been found to lag behind weight gain by approximately 3 months in undernourished children, emphasizing the importance of intervention durations of at least 120–180 days to adequately assess effects on linear growth [13]. Most successful studies examining height gain in stunted children have employed interventions lasting 6 months or longer [13,23].

### 1.5. Evidence Gap

Despite the growing body of evidence from neighboring countries such as Vietnam, India, and other Asian settings, evidence specific to Malaysia remains critically limited, particularly in regions such as Kelantan where the burden of stunting is highest [6]. No community-based intervention trials examining the efficacy of nutrient-dense ONS have been conducted in Malaysian populations. This gap is particularly significant given Malaysia’s unique stunting profile, which differs from typical patterns observed in other countries, where stunting correlates strongly with poverty and low parental education. The lack of locally generated evidence limits the ability of policymakers to design and implement context-appropriate interventions that address the specific determinants of stunting in Malaysia.

Furthermore, while international evidence demonstrates the efficacy of ONS in children with severe stunting (HAZ < −2 SD), there is limited evidence on the effectiveness of such interventions in children who are at risk of stunting (HAZ between −2 and −1 SD)—a population that represents a critical window for preventive intervention before irreversible growth faltering occurs. This preventive approach aligns with WHO recommendations emphasizing early identification and intervention for children at nutritional risk [2].

### 1.6. Study Objectives

The present study was designed to address these critical evidence gaps by evaluating the efficacy of nutrient-dense ONS on linear and ponderal growth among Malaysian children aged 12–36 months who are stunted or at risk of stunting. Specifically, this study aimed to (1) determine whether daily consumption nutrient-dense ONS for 180 days improves stunting in children with a compromised nutritional status; (2) estimate the rate of stunting, underweight, wasting, and overweight among the study children at baseline and subsequently at 90 days and at 180 days after intervention; and (3) determine the changes in nutrient intake before and after the intervention.

### 1.7. Study Significance and Expected Impact

The findings will provide locally generated evidence to inform policy development, clinical practice guidelines, and program scalability for nutrition interventions. By including both stunted and at-risk children during the critical 12–36-month age window—when growth failure is potentially reversible—this research addresses both treatment and prevention within a single framework. The evidence generated from this trial would provide a scalable, evidence-based intervention that could be integrated into Malaysia’s existing maternal and child health programs to reverse the alarming trend of increasing childhood stunting in the country.

## 2. Materials and Methods

This study was conducted in Kelantan, Malaysia from December 2022 to March 2024 by using a community-based single-arm intervention trial design.

### 2.1. Eligibility Criteria

Eligible children were Malaysian citizens, boys or girls aged between 12 and 36 months, with a HAZ between <−1.0 SD and >−3 SD and a weight-for-age z-score (WAZ) between <−1.0 SD and ≥−3 SD, according to the WHO Child Growth Standards. Parents or legal guardians were required to be able to read and communicate in either Malay or English and to provide written informed consent prior to participation. Only apparently healthy children with no current infection, chronic illness, or physical or mental disability were included. In addition, parents or legal guardians were required to own a mobile or smartphone, and mothers were required to have previously received breastfeeding counseling from a qualified breastfeeding counselor.

Exclusion criteria included children with any chronic disease, congenital disorder, or deformity. Children with an ongoing episode of diarrhea or a history of persistent diarrhea within the past month were excluded. Those with a known cow’s milk allergy or milk intolerance, children already consuming multivitamins (including iron) prior to enrolment, and those who were still receiving breast milk as part of their diet were also excluded.

### 2.2. Ethical Procedures

The protocols and materials of the present study were reviewed and approved by the Human Research Ethics Committee of Universiti Sains Malaysia (USM/JEPeM/22050302). This study was conducted in accordance with the ethical principles mentioned in the Declaration of Helsinki and was consistent with good clinical practice (GCP). This study was registered at clinicaltrials.gov (NCT05670002, 12 March 2025).

### 2.3. Screening and Recruitment of Subjects

Children were identified from 50 childcare centers (nurseries) in Kelantan using a convenience sampling approach. This community-based study was conducted in nurseries located in areas surrounding the Universiti Sains Malaysia (USM) campus. The nurseries were selected based on their proximity to the USM campus and their willingness to participate.

Of the 1438 children assessed for eligibility, 118 met the inclusion criteria and were enrolled after obtaining parental consent, and 91 children completed this study.

### 2.4. Sample Size Calculation

**Figure 1:**
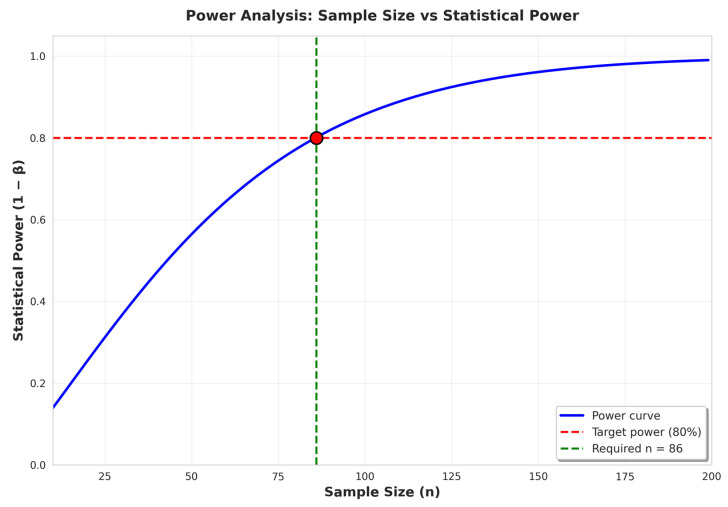
Power analysis curve.

The power analysis for the primary outcome was based on a published report [26]. Baseline and post-intervention height-for-age z-scores (HAZs) were −1.96 ± 0.62 and −1.70 ± 0.76, respectively. The estimated mean difference (effect size) between pre- and post-test was 0.30, with a pooled standard deviation of 0.98. Assuming a 95% confidence level (α = 0.05) and 80% power (β = 0.20), the required sample size was 86. Considering a 28% dropout rate, the final target sample size was 110 participants. A power analysis curve is shown in Figure 1.

### 2.5. Anthropometric Measurements

Anthropometric assessments were conducted at three time points: baseline (T0), day 90 (T90; mid-intervention), and day 180 (T180; post-intervention).

Standing height was measured in duplicate using a SECA stadiometer, recorded to the nearest 0.1 cm. Children were measured without hair ornaments, headgear, or jewelry. During measurement, each child stood barefoot on the stadiometer platform with feet together, body upright, arms relaxed at the sides, and the head positioned in the Frankfurt horizontal plane. If the discrepancy between the two measurements exceeded the maximum allowable limits, a third measurement was obtained, and the median value was recorded. The maximum allowable difference was defined as <0.1 kg for body weight and <0.5 cm for height. BMI was calculated by dividing body weight (kg) by height squared (m^2^). Anthropometric status was classified using the WHO Anthro software (version 3.2.2) for children under five years of age and standardized for sex and age based on the WHO (2006) reference standards [2]. Stunting was defined as HAZ < −2 SD, at risk of stunting as HAZ between −2 SD and <−1 SD, and normal height growth as HAZ ≥ −1 SD [2]. HAZ scores were calculated using raw anthropometric measurements.

Body weight was measured in duplicate using an automatic digital scale (SECA 769) with a precision of 10 g. Measurements were taken on a flat, stable surface with children in minimal clothing and without shoes or jewelry. If duplicate measurements differed by more than 10%, a third measurement was obtained, and the mean of the closest values was calculated. The scale was calibrated at each measurement session. According to WHO (2006) reference standards, underweight was defined as WAZ between −3 SD and <−2 SD, and normal weight as WAZ between −2 SD and 2 SD. Wasting was defined as a weight-for-height z-score WHZ between −3 SD and <−2 SD, overweight as WHZ > 2 SD to 3 SD and normal as −2 SD to 2 SD [2].

### 2.6. Dietary Assessment

A quantitative 3-day, 24 h dietary recall including two weekdays and one weekend day was conducted at baseline (T0) and post-intervention (T180) to estimate energy and nutrient intakes. The first recall day was administered in person during kindergarten visits with the parent or caregiver, while the remaining two recall days were conducted via telephone. Interviews were conducted by trained research assistants under the supervision of a nutritionist and were carried out without prior notification to the mothers. A standardized probing guide was used to facilitate recall of foods and beverages that are commonly overlooked or forgotten. Household measures and food photographs from the *Malaysia Food Album (Album Makanan Malaysia)* [27] were used as portion-size estimation aids during both in-person and telephone interviews, with a soft copy of the household measurement booklet and food album provided to participants for reference during telephone interviews.

In general, this study did not interfere with participants’ usual dietary practices. The children continued their habitual diets throughout the intervention, and no dietary modifications or restrictions were advised.

Nutrient analysis and the total energy intake calculations (kcal/day) were performed using Nutritionist Pro™ Diet Analysis version 7.8.0 software, developed by (Axxya Systems, version 2020, Redmond, WA, USA).

Recipes that were not available in the reference database were manually entered into the software. Portion sizes, were calculated based on standard recipe sizes, for example per serving size and total serving. The weight of foods and ingredients used in recipe preparation were sourced primarily from the Nutrient Composition of Malaysian Foods [28], supplemented by the USDA database (U.S. Department of Agriculture, Agricultural Research Service (USDA ARS, 2024)), [29] and the Malaysian Food Album (Album Makanan Malaysia) [27]. Nutritional information for commercial food products was obtained from product labels and packaging or from the Malaysian Food Composition Database (MyFCD) [30], and subsequently entered into the database.

Prior to analysis, plausibility of intake was evaluated using the formula of Black and Cole (2000) [26], by calculating the ratio of reported energy intake (EI) to predicted energy expenditure (EE). EE was estimated as the basal metabolic rate (BMR) [31,32] multiplied by a physical activity level (PAL) of 1.60, assuming moderate activity for children aged 12–48 months [33]. Following Black and Cole (2000) and using a 99% CI, within-subject coefficients of variation of 8.2% for EE and 23% for EI were applied [34].

$$99\%\;\mathrm{CI}\;=\pm 3\times \sqrt{\left(\frac{{{\mathrm{CV}}_{\mathrm{wEI}}}^{2}}{d}+\left({{\mathrm{CV}}_{\mathrm{wEE}}}^{2}\right)\right)}$$

where CV_wEE_ = within-subject variation in EE, 8.2%, CV_wEI_ = within-subject variation in EI, 23%, and *d* = number of days of diet assessment.

The Scofield equation [31] was used for estimating BMR in kcal/day (kilocalories per day) from body weight (kg).

The acceptable EI:EE range was 0.30–1.70; only children within this range were retained for dietary analyses. Out of the 91 children, 4 overreporting subjects were excluded and a total of 87 children were included in the analysis. Nutrient analyses did not include intake from dietary supplements.

For each participant, energy intake and macro- and micronutrients were averaged across the three 24 h dietary recalls (two weekdays and one weekend day). The reported total energy represents the mean daily energy intake across all 87 subjects included in the analysis.

### 2.7. Study Intervention

All subjects were asked to consume 510 mL of a nutrient-dense oral nutrition supplement (ONS) daily (170 mL × 3 servings/day) for a duration of 180 days (6 months). Subjects were asked to take the product, three servings per day: (1) in the morning, upon waking, (2) 2–3 h after lunch and (3) before going to bed at night. Preparation for each serving: 1 sachet (27 g) of ONS powder + 150 mL water = 170 mL.

### 2.8. ONS Delivery and Subject Compliance

The oral nutrition supplement (ONS) was distributed in a staged manner: a one-month supply at baseline, a two-month supply at 30 days, and a three-month supply at 90 days, which is a total of 180 days’ supply that was delivered through home or nursery visits. To ensure compliance, parents were contacted by trained research assistants via telephone at 60, 120, and 150 days after the start of the intervention. Research assistants maintained follow-up calendars for each child and ensured that supplements were delivered before the existing supply was depleted.

Subjects’ compliance was assessed by collecting and counting the number of empty sachets returned. Compliance at each time point was calculated as the number of sachets returned relative to the total number of sachets provided, multiplied by 100 to find the percentage. The overall compliance percentage was determined by averaging values across all the three time points. The subject was considered compliant when the average percentage of all time points is 80% and above. The mean compliance rate among all children was 97.4% ± 6.7 and all children met the predefined compliance threshold of ≥80%.

### 2.9. Data Management and Statistical Analysis

Data quality was assessed in two steps: First, all data entered into Microsoft SharePoint were verified for accuracy with the paper forms and questionnaires. Next, the data entered into Microsoft SharePoint were transferred for data cleaning and analysis using IBM SPSS Statistics for Windows, version 29.0 (IBM Corp., Armonk, NY, USA). The computer was a password-protected institutional platform, accessible only to authorized members of the research team. During the data cleaning process, logic checks were performed to identify inconsistencies and implausible values between related variables (e.g., age versus date of birth, date of measurement, consistency of dietary entry, etc.). Implausible or inconsistent data were verified against source forms and corrected when necessary. Improvements in linear growth were evaluated by comparing height, weight, body mass index (BMI), height-for-age z-score (HAZ), weight-for-age z-score (WAZ), and weight-for-height z-score (WHZ) at three time points: baseline (T0), 90 days (T90), and 180 days (T180). Data were first assessed for normality. Normally distributed variables were analyzed using repeated-measures analysis of variance (ANOVA), whereas non-normally distributed variables were analyzed using the Friedman test.

Comparisons of HAZ and WAZ between stunted and at-risk groups across the three time points were also performed. Post hoc analyses were conducted to identify significant differences between time points; Tukey’s honestly significant difference (HSD) test was applied for normally distributed data, and the Wilcoxon signed-rank test was used for non-normally distributed data.

Changes in the distribution of height-for-age (HAZ) categories (stunted and at-risk) over time were analyzed using the McNemar test. Changes in nutrient intakes, including energy (kcal), protein (g), carbohydrate (g), fat (g), vitamins (mg or µg), and minerals (mg or µg), between baseline and 180 days were first assessed for normality; paired-sample *t*-tests were used for normally distributed data, and the Wilcoxon signed-rank test was applied for non-normally distributed data. Statistical significance was defined as *p* ≤ 0.05.

## 3. Results

**Figure 2:**
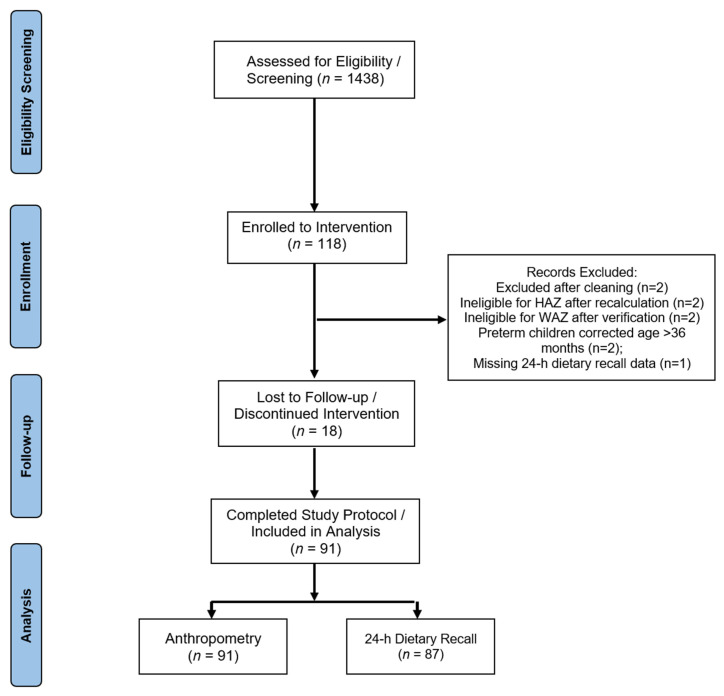
Study CONSORT flowchart.

Figure 2 shows that a total of 1438 children were screened and assessed for eligibility, of whom 118 were enrolled in this study. Following enrolment and data cleaning, nine children were excluded from the analysis. Of these, two did not meet the inclusion criteria for height-for-age z-score (HAZ) after recalculation, two were preterm children whose corrected age exceeded 36 months, four did not meet the inclusion criteria for weight-for-age z-score (WAZ) after data verification, and one child had missing 24 h dietary recall data. In addition, 18 children were lost to follow-up or discontinued the intervention. Ultimately, 91 children completed this study and were included in the final analysis.

### 3.1. Sociodemographic Profile

Table 1 shows that all participants were Malay children, and were Muslims, because the study area, Kelantan, is a Malay-predominating Islamic state [35]. Among the enrolled children, approximately two-thirds (64.8%) were boys, whereas 35.2% were girls. The male predominance of the sample mirrored the normal admission pattern in the nurseries from where the samples were drawn. No gender-based stratification or restrictions were applied during recruitment. The majority of children attended nursery (83.5%), but only 3.3% went to kindergarten or preschool. Almost all parents or guardians were married (98.9%). The majority of participants’ fathers had tertiary education (73.6%), while 23.1% had completed secondary education, and 2.2% had basic education or no formal schooling. Similarly, most of the mothers had tertiary education (78.0%). The data were comparable with the characteristics of the population of Kelantan, Malaysia [35].

### 3.2. Anthropometric Results

**Figure 3:**
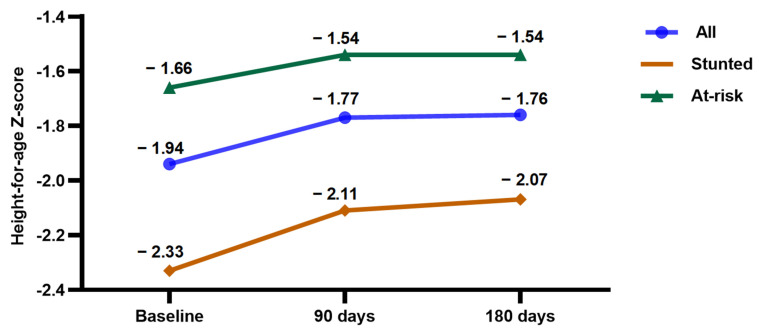
Height-for-age z-score (HAZ) changes after intervention.

HAZ scores are presented in the three groups—stunted, at-risk stunting and all children at baseline and after intervention in Figure 3. Changes in HAZ show that HAZ increased from a baseline of −1.94 ± 0.42 to −1.77 ± 0.43 at 90 days, and to −1.76 ± 0.41 at 180 days. Post hoc tests confirmed significant increases between baseline and 90 days (*p* < 0.001) and baseline and 180 days (*p* < 0.001), with no statistically significant differences observed between 90 and 180 days.

When comparing HAZ between groups (stunted vs. at-risk), a significant group effect was observed (*p* < 0.001). In the stunted group, HAZ improved from −2.33 ± 0.29 at baseline to −2.11 ± 0.38 at 90 days and −2.07 ± 0.44 at 180 days. In the at-risk group, HAZ improved from −1.66 ± 0.24 at baseline to −1.54 ± 0.29 at 90 days and −1.54 ± 0.27 at 180 days. Post hoc analysis showed significant increases for both groups from baseline to 90 days and baseline to 180 days (all *p* < 0.001). From 90 to 180 days, significant improvement was seen only in the stunted group (*p* < 0.001), while no change was detected in the at-risk group (*p* > 0.05).

Table 2 summarizes the anthropometric indicators at baseline (T_0_), 90 days, and 180 days. Height increased significantly over time (*p* < 0.001), from baseline 84.2 ± 4.91 cm to 86.7 ± 4.58 cm at 90 days and to 88.7 ± 4.26 cm at 180 days. Post hoc analysis confirmed significant differences across all pairwise comparisons (baseline vs. 90 days, baseline vs. 180 days, and 90 days vs. 180 days; all *p* < 0.001).

Weight also showed a significant main effect of time (*p* < 0.001)—children’s mean weight increased from a baseline of 11.1 ± 1.24 kg to 11.6 ± 1.28 kg at 90 days, and to 12.2 ± 1.36 kg at 180 days. Post hoc analysis indicated a significant gain between the baseline and 180 days (*p* < 0.001), whereas changes between the baseline and 90 days and between 90 and 180 days were not statistically significant.

For BMI, a significant main effect of time was observed (*p* < 0.01). Mean BMI values decreased slightly from 15.7 ± 1.23 kg/m^2^ at baseline to 15.5 ± 1.19 kg/m^2^ at 90 days and remained stable at 15.5 ± 1.26 kg/m^2^ at 180 days.

WAZ changed significantly over time and differed between nutritional status groups (*p* < 0.001). In the stunted group, mean WAZ increased modestly from −1.58 ± 0.60 at baseline to −1.57 ± 0.65 at 90 days and −1.49 ± 0.70 at 180 days, with no significant differences between time points. In contrast, the at-risk group showed a significant improvement in WAZ from −1.13 ± 0.67 at baseline to −1.04 ± 0.63 at 90 days and −1.01 ± 0.70 at 180 days (*p* < 0.05), with no difference between 90 and 180 days.

WHZ did not change significantly across time points. Mean WHZ values were −0.40 ± 0.85, −0.43 ± 0.86 and −0.34 ± 0.95 at baseline, 90 days and 180 days, respectively.

Table 3 presents the percentage of children by nutritional status indicators (HAZ, WAZ, and WHZ) across baseline, 90 days, and 180 days. The percentage of stunted children decreased from 40.7% at baseline to 33.0% at 90 days and to 25.3% at 180 days. At the same time, the proportion of children classified as at risk of stunting increased from 59.3% to 65.9% and 73.6% at 90 days and at 180 days, respectively. One child (1.1%) achieved a normal HAZ status at both 90 and 180 days. The McNemar test indicated a statistically significant reduction in stunting of nearly 38%, from baseline to 180 days (*p* = 0.003).

For underweight status, the prevalence decreased from 14.3% at baseline to 13.2% at 90 days and to 8.8% at 180 days, accompanied by an increase in the proportion of children with normal weight-for-age z-scores (from 85.7% to 91.2%). Although these changes reflect a positive shift in nutritional status, the differences were not statistically significant.

Wasting remained stable at 3.3% from baseline to 90 days before falling to 1.1% at 180 days. Two children (2.2%) were classified as overweight at 180 days. These latter two children were already near the upper cut-off at baseline, and their classification shift is consistent with expected growth patterns rather than disproportionate increases in WHZ.

### 3.3. 24 h Dietary Recall

Table 4 summarizes changes in nutrient intake across the baseline and 180 days. Overall, significant improvements were observed in almost all macronutrients and micronutrients when comparing the baseline to 180 days, except carbohydrates. This indicates that while the overall dietary profile improved, carbohydrate intake remained relatively stable throughout the intervention. In general, participants’ habitual diets were kept constant throughout the intervention, as no dietary modifications or restrictions were imposed. This study did not interfere with participants’ usual dietary practices.

**Figure 4:**
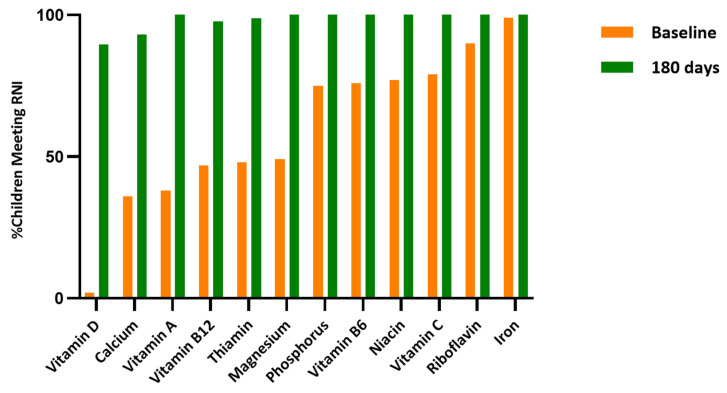
Percentage of children that met RNI at baseline and at 180 days.

Figure 4 shows the percentage of children meeting the level of the Malaysian Recommended Nutrient Intake (RNI) at baseline and at 180 days. At baseline, adequacy varied widely across nutrients: iron (99%), riboflavin (90%), vitamin C (79%), and niacin (77%) were relatively high, whereas fewer than half of the children met the RNI levels for calcium (36%), vitamin A (38%), vitamin B12 (47%), thiamine (48%), and magnesium (49%). Vitamin D adequacy was the lowest, with only 2% of children meeting the RNI level.

By 180 days, notable improvements were observed across nearly all nutrients, with adequacy approaching or reaching 100% for most vitamins and minerals. Calcium increased from 36% at baseline to 91% at 180 days, while vitamin D rose from 2% to 89%. Thiamine (99%) and vitamin B12 (98%) also improved markedly, though they remained slightly lower than other nutrients. Overall, the intervention resulted in a substantial shift from suboptimal to adequate intake levels across almost all micronutrients.

## 4. Discussion

This community-based intervention study demonstrates that 510 mL daily of nutrient-dense oral nutrition supplementation (ONS) substantially improved growth and the nutritional status among stunted and at-risk Malaysian children aged 12–36 months. Over 180 days, participants showed significant increases in height, weight, height-for-age z-score (HAZ) and weight-for-age-z-score (WAZ), with improvements evident as early as 90 days in stunting. By the end of the intervention, there was a reduction of nearly 38% of the number of stunted children, representing a clinically meaningful shift in nutritional status within this vulnerable population. Two cases reported a borderline overweight status at the end of this study, but these children were already near the cut-off at baseline, and the results reflect minor shifts across anthropometric thresholds rather than excessive or abnormal weight gain, suggesting that the intervention maintained appropriate growth trajectories without promoting overnutrition.

### 4.1. Comparison with Malaysian Studies

To our knowledge, this is the first community-based ONS intervention trial specifically targeting stunted and at-risk children in Malaysia, particularly in Kelantan, the state with the highest stunting burden (28.8%) [36]. Previous Malaysian research has predominantly focused on identifying the determinants and prevalence of stunting rather than evaluating nutritional interventions [37,38]. A recent cross-sectional study in Terengganu among children under 2 years found that 19.3% were stunted, with low birth weight and poor maternal nutritional knowledge identified as key risk factors, though no intervention was tested [39]. A nationwide survey across 11 states found that approximately one in five children reported at least one form of undernutrition, emphasizing the need for evidence-based nutritional intervention strategies [40].

Our study addresses this critical evidence gap by providing the first locally generated data on ONS efficacy in the Malaysian context. The 38% reduction in stunting percentage within 6 months is particularly encouraging given that Malaysia’s Deputy Health Ministry reported that almost 30% of young children suffer from stunted growth, with Kelantan having among the highest rates [7]. This finding is especially relevant to Malaysia’s policy context, as the country’s stunting rate has increased significantly from 17.7% in 2015 to 21.2% in 2022, far exceeding the National Plan of Action for Nutrition target of 14.2% by 2030 [5,41]. Our results suggest that ONS interventions could be a viable component of Malaysia’s multisectoral strategy to reverse this alarming trend.

Importantly, ONS should not be viewed as a replacement for optimal maternal nutrition and recommended infant and young child feeding practices, but rather as a complementary approach in contexts where fundamental feeding gaps persist. In the present study, continued breastfeeding beyond infancy was not practiced, as children who were still receiving breast milk were excluded, despite World Health Organization recommendations to continue breastfeeding up to two years of age and beyond. In addition, qualitative assessment of dietary intake indicated a suboptimal diet quality among participating children, characterized by limited dietary diversity and insufficient intake of key macro- and micronutrients essential for linear growth. In such settings, where improvements in maternal nutrition, breastfeeding continuation, and complementary feeding quality may be constrained by socioeconomic and behavioral factors, the addition of a nutritionally complete ONS may help bridge existing nutrient gaps and support catch-up growth in at-risk and stunted children.

### 4.2. Comparison with Neighboring Country Data

Our findings align closely with recent evidence from neighboring Asian countries. In a prospective multicenter trial in India, ONS with dietary counseling in children aged 24–48 months, who were at risk of malnutrition (weight-for-height percentile 3rd to 15th) found significant improvements in weight-for-height percentile in the intervention group, as compared to dietary counseling alone (*p* = 0.009). Anthropometric measurements such as weight and body mass index also increased significantly in the intervention group [23]. Similarly, Pham et al. (2019) demonstrated that six months of ONS improved HAZs and weight-for-age z-scores (WAZs), with approximately 40% of stunted Vietnamese children recovering to a normal height status [24]. Our study’s finding of a significant HAZ improvement within 180 days parallels these Vietnamese results, despite our slightly younger cohort (12–36 months versus 24–60 months).

A recent randomized controlled trial in India involving 223 children aged 3.0 to 6.9 years found that those receiving daily ONS plus dietary counseling grew taller and stronger than those receiving counseling alone [25]. Earlier Indian studies by Sazawal et al. (2010) reported parallel improvements in HAZs, WAZs, and weight-for-height z-scores (WHZs) following ONS intervention [42]. Another randomized controlled study from India among children at risk of malnutrition (weight-for-height between the 3rd and 15th percentiles) demonstrated that the addition of ONS to standard dietary counseling— consisting of general caregiver advice on age-appropriate balanced diets and healthy feeding practices—resulted in greater improvements in nutrient intake and growth indicators compared with dietary counseling alone [43]. Ghosh et al. (2018) conducted a 90-day randomized controlled trial in India showing an improved nutrient intake and growth, though height changes were modest over the short duration, underscoring the importance of our 180-day intervention period [43].

Studies from other Asian settings further corroborate our findings. Huynh et al. (2015, 2016) in Philippines reported steady increases in height and weight over 48 weeks of ONS in 3–4-year-olds, with compliance above 80% [44,45]. Devaera et al. (2018) in Indonesia tested ONS over 28 days and observed weight gain but no significant differences in height between groups, reinforcing that longer intervention durations are essential to detect linear growth changes [46]. Similarly, in a recent Indonesian cluster randomized controlled trial by Chandra et al. (2025), children who received 400 mL/day of a fortified young child “growth milk”—a milk-based product enriched with energy, protein, and essential micronutrients—for 3 months demonstrated a significant increase in HAZ from −1.65 to −1.58 in the intervention group, indicating a modest but positive effect of “growth milk” supplementation on linear growth [47]. Collectively, these Asian studies support our conclusion that ONS, when delivered consistently at an adequate dosage (typically ≥400–600 mL/day) with appropriate clinical monitoring, can reverse early growth faltering across diverse Asian populations.

The dose–response relationship appears important. Evidence from Nigeria demonstrated that only the highest ONS intake (600 mL/day) improved HAZ, while lower doses showed benefits primarily for weight [26]. Our study provided 510 mL/day, which appears to fall within the effective range for promoting both linear and ponderal growth.

### 4.3. Comparison with Global Evidence

Our findings contribute to the broader international evidence base on ONS efficacy for childhood undernutrition. Recent systematic reviews and meta-analyses have confirmed that polymeric ONS providing complete blends of macronutrients and micronutrients is effective in promoting growth in children aged 9 months to 12 years with undernutrition [48,49]. Multiple micronutrient formulations have been found more effective than single-nutrient approaches, as poor growth in many developing and developed countries commonly results from multiple nutrient deficiencies rather than single deficiencies [50].

The pattern of weight gains preceding height gains observed in our study is consistent with global evidence. Previous randomized controlled trials have demonstrated improved weight gain as early as 10 days and consistently at 30 days, while results for height gain are less consistent in short-duration trials [13,51]. Among 90-day randomized controlled trials, height gain was significantly larger in the ONS group in only one trial and trended larger but non-significant in two others [52,53]. However, a systematic review and meta-analysis showed that in studies with longer durations (≥180 days), the nutritional interventions significantly improved the weight-for-height z scores compared to studies where the nutritional intervention period was shorter than 180 days [54]. Our 180-day intervention duration was therefore appropriately designed to capture linear growth changes.

### 4.4. Age Considerations for Linear Growth

Our study’s finding of significant improvement in stunted children within 180 days is particularly encouraging given that participants were at least 12.9 months old at baseline (mean age: 27 months). This supports previous longitudinal analyses demonstrating that linear catch-up growth can occur beyond 24 months or after the first 1000 days of life [55]. While the effects of stunting are largely irreversible after the first 1000 days, and school-level interventions may not impact stunting itself [56], our data suggest that targeted nutritional interventions during the 12–36-month period—which extends slightly beyond the traditional 1000-day window—can still produce meaningful improvements in linear growth. This finding has important programmatic implications, as it suggests that children identified with stunting or an at-risk status during routine growth monitoring beyond 24 months may still benefit from intensive nutritional support.

From a programmatic perspective, these findings underscore the potential value of identifying and supporting children with stunting or an at-risk status through routine growth monitoring beyond 24 months of age. The sustained improvement in HAZ observed between day 90 and day 180 exclusively in the stunted group may reflect a greater capacity for catch-up growth among children with more pronounced baseline growth deficits. In contrast, children at risk of stunting, who were closer to expected growth trajectories at baseline, may require longer intervention durations or additional complementary strategies to achieve further linear growth gains.

Finally, although we observed a statistically significant reduction in BMI, this change should be interpreted cautiously, as it likely reflects proportional increases in height relative to weight gain rather than a clinically meaningful improvement in adiposity.

### 4.5. Mechanistic Considerations

The catch-up growth observed in our study can be explained by the synergistic roles of key nutrients in skeletal development and overall growth. Bone elongation occurs at the epiphyseal growth plate, where chondrocytes proliferate, mature, and ossify, driving longitudinal bone growth [57]. An adequate protein intake is essential to meet amino acid demands for tissue synthesis and to stimulate growth-promoting hormones. Dietary protein raises insulin and insulin-like growth factor-1 (IGF-1), both of which enhance chondrocyte activity and bone matrix formation [58].

Alongside protein, calcium, phosphorus, magnesium, vitamin D, and vitamin K are required for optimal bone mineralization and structural integrity [59]. Deficiencies in any of these nutrients can impair linear growth and compromise bone health [60]. The ONS used in our study provided 360 kcal per day, 16.2 g protein, 13.8 g fat, and 42 g carbohydrates, along with a comprehensive micronutrient profile including calcium (753 mg), iron (6 mg), zinc (4.5 mg), vitamin D3 (12.9 mcg), vitamin C (93 mg), and B vitamins. This complete nutritional package addresses both macronutrient energy requirements and micronutrient needs for growth.

An important consideration is the potential for a “wash-out” effect following cessation of ONS at day 180. Although the present study demonstrated significant improvements in linear growth during the intervention period, it is unclear whether these gains would be sustained after the supplementation has been discontinued. The persistence of improved growth trajectories is likely influenced by concurrent changes in feeding practices, dietary quality, and caregiving behaviors. In contexts where habitual diets remain nutritionally inadequate, discontinuation of ONS may limit continued linear growth, particularly among children exposed to persistent dietary and socioeconomic constraints. Conversely, ONS may function as a time-limited nutritional support that facilitates partial catch-up growth and enhances growth potential, even if subsequent gains diminish after intervention withdrawal. Accordingly, longer-term follow-up studies are needed to assess the durability of growth responses and to determine whether combining ONS with sustained improvements in complementary feeding and broader nutrition-sensitive interventions can reduce potential wash-out effects.

### 4.6. Study Strengths

This study employed standardized WHO anthropometric assessment methods and included both stunted (HAZ < −2 SD) and at-risk children (HAZ between ≥ −2 SD and < −1 SD), providing evidence on both treatment and prevention strategies within a single intervention framework [61]. This dual approach is pragmatically important, as identifying and intervening with at-risk children may prevent progression to stunting.

Second, the 180-day intervention duration was appropriately designed based on evidence that linear growth lags behind weight gain by approximately 3 months in undernourished children, ensuring adequate time to observe changes in HAZ. The detection of improvements as early as 90 days also provides valuable information on the time course of nutritional recovery.

### 4.7. Study Limitations

We acknowledge several important limitations that should be considered when interpreting our findings.

The most significant limitation is the absence of a control group. While ethical considerations informed this decision, as withholding a potentially beneficial nutritional intervention from already stunted or at-risk children raised ethical concerns, this design limits our ability to definitively attribute the observed improvements solely to the ONS intervention. Our study also had a limitation that the child growth was not controlled for maternal nutrition. However, a recent controlled study reported by Khadilkar et al. (2025) in India [25] already demonstrated that ONS with nutritional counseling had significantly greater benefits in improving anthropometric scores compared with counseling alone.

While our study demonstrated clinically meaningful improvements, the sample size may have limited our ability to detect differential effects across subgroups (e.g., by severity of stunting, age, sex, or socioeconomic status). Larger sample sizes in future studies would enable more robust subgroup analyses to identify which children benefit most from ONS interventions.

The use of parent-reported 24 h dietary recalls is subject to recall bias, social desirability bias, and potential underreporting or overreporting of food intake. While 24 h recalls are widely used and validated tools for dietary assessment, more objective measures such as biomarkers of nutritional status (serum micronutrient levels, hemoglobin, albumin) would strengthen future studies.

To minimize potential social desirability bias in caregiver-reported supplement intake, several measures were implemented. Compliance was monitored through multiple approaches, including collection of empty sachets, regular caregiver calls conducted by trained study personnel using neutral, non-judgmental questioning, and cross-checking reported intake with scheduled distribution records. Caregivers were informed that reporting non-consumption would not result in penalties or withdrawal from this study, thereby reducing pressure to overreport adherence. Despite these measures, some degree of reporting bias cannot be fully ruled out.

## 5. Conclusions

This community-based intervention study provides the first Malaysian evidence that daily consumption of 510 mL (three 170 mL servings) of a nutrient-dense oral nutrition supplement over 180 days significantly improved linear and ponderal growth in children aged 12–36 months who were stunted or at risk of stunting. Significant gains in height and weight were observed as early as 90 days, with sustained improvements through 180 days. Clinically, the intervention achieved an approximate 38% reduction in stunting prevalence within 6 months, representing a meaningful shift in nutritional status within this high-burden population.

To reiterate, the study results should be measured with caution in light of a major limitation of the absence of a control group. It is also emphasized that future studies should address maternal nutrition as a factor of the child growth and nutrition. A well-controlled, randomized trial, with adequate nutrition programs for women, in addition to ONS, is recommended for future studies.

These findings are particularly significant given that Malaysia is experiencing an alarming increase in childhood stunting rates, contrary to declining trends in neighboring Southeast Asian nations, and is currently off-track to meet national targets.

Overall, these findings highlight the potential of fortified ONS as a core component of evidence-based strategies to address stunting and associated micronutrient deficiencies in Malaysian children aged 12–36 months. Despite several limitations, our study provides a crucial proof of concept that targeted nutritional supplementation can produce clinically significant improvements in growth outcomes among Malaysia’s most nutritionally vulnerable children.

As Malaysia intensifies efforts to reverse rising stunting trends and meet national and global nutrition targets, the evidence generated from this study offers a viable, scalable intervention that can be implemented while more comprehensive, multisectoral approaches are developed and tested. The findings support calls for evidence-based nutrition policies in Malaysia and contribute to the regional knowledge base on effective stunting interventions in middle-income Southeast Asian countries facing the paradox of rising child undernutrition amid economic development.

## Figures and Tables

**Table 1:** Characteristics of the study participants.

Variables	n	(%)	Mean ± SD	Range [Min, Max]	95% CI
Sex					
Male	59	64.8			
Female	32	35.2			
Age (Months) (during Recruitment at Baseline)			26.7 ± 6.6	[12.9, 36.0]	[25.2, 28.0]
12–23.99	28	30.8			
24–36	63	69.2			
Premature Birth Before 37 Weeks of Pregnancy					
Yes	4	4.4			
No	87	95.6			
Race					
Malay	91	100.0			
Religion					
Islam	91	100.0			
Attending Kindergarten/Pre-School					
Yes	3	3.3			
No	88	96.7			
Attending Nursery					
Yes	76	83.5			
No	15	16.5			
Current Parents’/Guardians’ Marital Status					
Married	90	98.9			
Separated/Divorced	1	1.1			
Father Highest Educational Level ^1^					
Non-Schooling/Primary School	2	2.2			
Secondary School	21	23.1			
Tertiary School (College/University)	67	73.6			
Mother Highest Educational Level					
Secondary School	20	22.0			
Tertiary School (College/University)	71	78.0			
Approximate Total Monthly Household Income (Kelantan) ^2^			6080.48 ± 4640.28		[5114.10, 7046.87]
B40 (MYR < 3060)	26	28.6			
M40 (MYR 3061–6469)	34	37.4			
T20(MYR 6470 and above)	31	34.1			

^1^ Missing = 1. ^2^ Source: Household Income Survey Report 2022: Technical Notes, Department of Statistics Malaysia; USD 1 dollar = MYR 4.09 (as of 16 December 2025); B40: low income; M40: middle income; T20: high income.

**Table 2:** Anthropometric results (height, weight, BMI, HAZ, WAZ and WHZ).

Variable	Categories	N	T_0_ (Baseline)	T = 90 Days	T = 180 Days	*p*-Value
Height (cm) *		91	84.2 ± 4.91 ^a^	86.7 ± 4.58 ^b^	88.7 ± 4.26 ^c^	<0.001 ^d^
Weight (kg) **		91	11.1 ± 1.24 ^a^	11.6 ± 1.28 ^b^	12.2 ± 1.36 ^c^	<0.001 ^e^
BMI (kg/m^2^) **		91	15.7 ± 1.23 ^a^	15.5 ±1.19 ^b^	15.5 ±1.26 ^b^	0.004 ^e^
HAZ **		91	−1.94 ± 0.42 ^a^	−1.77 ± 0.43 ^b^	−1.76 ± 0.41 ^b^	<0.001 ^e^
	Stunted	37	−2.33 ± 0.29 ^a^	−2.11 ± 0.38 ^b^	−2.07 ± 0.44 ^b^	<0.001 ^f^
	At-risk	54	−1.66 ± 0.24 ^a^	−1.54 ± 0.29 ^b^	−1.54 ± 0.27 ^b^	
WAZ **		91	−1.31 ± 0.68 ^a^	−1.26 ± 0.68 ^b^	−1.20 ± 0.74 ^b^	0.005 ^e^
	Stunted	37	−1.58 ± 0.60 ^a^	−1.57 ± 0.65 ^a^	−1.49 ± 0.70 ^a^	0.007 ^f^
	At-risk	54	−1.13 ± 0.67 ^a^	−1.04 ± 0.63 ^b^	−1.01 ± 0.70 ^b^	
WHZ		91	−0.40 ± 0.85	−0.43 ± 0.86	−0.34 ± 0.95	0.147 ^e^

HAZ, height-for-age z-score; WAZ, weight-for-age z-score; WHZ, weight-for-height z-score; BMI, body mass index. (*) ^a^, ^b^, ^c^: Post Hoc Wilcoxon Rank Test—significant differences between all-time points at *p* < 0.05. ^d^ Friedman Test—significant at *p* < 0.05. (**) ^a^, ^b^, ^c^: Repeated Measures ANOVA—post hoc Tukey’s HSD analysis showing significant differences between all time points at *p* < 0.05. ^e^ Repeated Measures ANOVA—significant difference between time points at *p* < 0.05. ^f^ Repeated Measures ANOVA—significant difference between groups (stunted vs. at-risk) at *p* < 0.05.

**Table 3:** Number and percentage of children with stunting, underweight, wasting, and overweight from baseline to 180 days.

	Baseline	90 Days	180 Days
Stunted	37 (40.7%)	30 (33.0%)	23 (25.3%)
At-risk	54 (59.3%)	60 (65.9%)	67 (73.6%)
Normal	-	1 (1.1%)	1 (1.1%)
Underweight	13 (14.3%)	12 (13.2%)	8 (8.8%)
Normal	78 (85.7%)	79 (86.8%)	83 (91.2%)
Wasting	3 (3.3%)	3 (3.3%)	1 (1.1%)
Normal	88 (96.7%)	88 (96.7%)	88 (96.7%)
Overweight	0	0	2 (2.2%)

Definition of nutritional status under 5 years old: HAZ, height-for-age z-score; WAZ, weight-for-age z-score; WHZ, weight-for-height z-score. Stunting: HAZ < −2 SD (at risk: −2 to <−1 SD); underweight: WAZ < −2 SD; wasting: WHZ < −2 SD; normal (most indices): −2 to +2 SD; overweight: >+2 SD.

**Table 4:** Nutrient intake (absolute values) at baseline and 180 days.

Nutrient	Baseline Mean ± SD	180 Days Mean ± SD	*p*-Value
Energy (Kcal)	1035.4 ± 208.2	1108.4 ± 161.0	0.012 ^a^
Protein (g)	40.2 ± 10.1	46.3 ± 7.8	<0.001 ^b^
Carbohydrate (g)	135.0 ± 35.9	139.4 ± 32.6	0.5082 ^b^
Fat, Total (g)	40.1 ± 12.9	43.4 ± 9.4	0.0079 ^b^
Thiamin (B1) (mg)	0.54 ± 0.27	0.82 ± 0.15	<0.001 ^a^
Riboflavin (B2) (mg)	1.1 ± 0.6	1.2 ± 0.2	0.0007 ^b^
Niacin (B3) (mg)	8.0 ± 2.6	11.6 ± 2.5	<0.001 ^b^
Vitamin B6 (mg)	0.7 ± 0.3	1.1 ± 0.3	<0.001 ^b^
Folate (mcg)	34.9 ± 25.0	40.8 ± 24.5	0.0274 ^b^
Vitamin B12 (mcg)	1.8 ± 1.3	2.9 ± 1.5	<0.001 ^b^
Vitamin C (mg)	55.8 ± 28.9	109.0 ± 24.8	<0.001 ^b^
Vitamin A (mcg)	377.8 ± 190.0	679.8 ± 145.4	<0.001 ^a^
Vitamin D (mcg)	6.1 ± 3.5	12.8 ± 2.3	<0.001 ^b^
Calcium (mg)	645.3 ± 214.2	886.3 ± 149.8	<0.001 ^b^
Iron (mg)	10.1 ± 3.2	12.0 ± 2.2	<0.001 ^b^
Phosphorus (mg)	608.5 ± 235.8	902.5 ± 149.7	<0.001 ^a^
Magnesium (mg)	81.1 ± 30.8	139.5 ± 20.5	<0.001 ^b^

Significant at *p* < 0.05. Statistical tests used: paired *t*-test (^a^) and Wilcoxon signed-rank test (^b^).

## Data Availability

Data generated or analyzed during this study are not publicly available due to confidentiality and compliance with European General Data Privacy Regulations. Data are, however, available from the authors upon reasonable request and with permission from the project funder.

## Abbreviations

BMI	Body Mass Index
CI	Confidence Interval
HAZ	Height-for-Age Z-Score
MYR	Malaysian Ringgit
NHMS	National Health and Morbidity Survey
ONS	Oral Nutrition Supplement
SD	Standard Deviation
USDA ARS	U.S. Department of Agriculture, Agricultural Research Service
WAZ	Weight-for-Age Z-Score
WHZ	Weight-for-Height Z-Score
WHO	World Health Organization
